# The effect of different boron compounds on nutrient digestibility, intestinal nutrient transporters, and liver lipid metabolism

**DOI:** 10.55730/1300-0144.5624

**Published:** 2023-04-15

**Authors:** Emre ŞAHİN, Cemal ORHAN, Füsun ERTEN, Fikrettin ŞAHİN, Nurhan ŞAHİN, Kazim ŞAHİN

**Affiliations:** 1 Department of Animal Nutrition, Faculty of Veterinary Medicine, Bingöl University, Bingöl, Turkey; 2 Department of Animal Nutrition, Faculty of Veterinary Medicine, Fırat University, Elazığ, Turkey; 3 Department of Veterinary Science, Pertek Sakine Genç Vocational School, Munzur University, Tunceli, Turkey; 4 Department of Genetics and Bioengineering, Faculty of Engineering and Architecture, Yeditepe University, İstanbul, Turkey

**Keywords:** Boron, energy metabolism, GLUTs, nutrient transporters, PPARγ

## Abstract

**Background/aim:**

Gastrointestinal health is essential for maintaining a healthy lifestyle. Improving nutrient absorption and energy metabolism are the critical targets for intestinal health. This study aimed to determine the effects of different boron (B) derivatives on nutrient digestibility, intestinal nutrient transporters, and lipid metabolism in rats.

**Materials and methods:**

Twenty-one rats were allocated to three groups (n = 7) as follows: (i) Control, (ii) Sodium pentaborate pentahydrate (SPP), and (iii) boric acid (BA). The rats were fed a chow diet (AIN-93M) and supplemented with 8 mg/kg elemental B from SPP (45.2 mg/kg BW) and BA (42.7 mg/kg BW) via oral gavage every other day for 12 weeks. The nutrient digestibility of rats in each group was measured using the indigestible indicator (chromium oxide, Cr_2_O_3_, 0.20%). At the end of the experiment, animals were decapitated by cervical dislocation and jejunum, and liver samples were taken from each animal. The nutrient transporters and lipid-regulated transcription factors were determined by RT-PCR.

**Results:**

The nutrient digestibility (except for ash) was increased by SPP and BA supplementation (p < 0.05). SPP and BA-supplemented rats had higher jejunal glucose transporter 1 (GLUT1), GLUT2, GLUT5, sodium-dependent glucose transporter 1 (SGLT1), fatty acid transport protein-1 (FATP1), and FATP4 mRNA expression levels compared to nonsupplemented rats (p < 0.0001). BA-supplemented rats had remarkably higher peroxisome proliferator-activated receptor gamma (PPARγ) levels than nonsupplemented rats (p < 0.0001). In contrast, sterol regulatory element-binding protein 1c (SREBP-1c), liver X receptor alpha (LxR-α), and fatty acid synthase (FAS) levels decreased by SPP supplementation compared to other groups (p < 0.05).

**Conclusion:**

SPP and BA administration enhanced nutrient digestibility, intestinal nutrient transporters, and liver lipid metabolism in rats.

## 1. Introduction

The worldwide prevalence and economic burden of gastrointestinal disorders (GID) are increasing rapidly [1]. One of the primary outcomes of the GID is the impairment of intestinal nutrient absorption ability [2]. Nutrient absorption is mediated by transporter proteins located on the intestinal epithelium’s brush border or basolateral membranes [3]. Carbohydrate absorption is mediated by the family of glucose transporters (GLUTs) and sodium-glucose cotransporters (SGLTs). SGLT1 and GLUT5 are vital in absorbing sodium-dependent glucose cotransporters and fructose for carbohydrate intake [4]. Basolaterally located, the monosaccharide transporter GLUT2 transports glucose, galactose, fructose, mannose, and glucosamine from the cell to the blood for carbohydrates [4]. Fatty acid transport protein-1 (FATP1) and FATP4, located in the endoplasmic reticulum of cells, control long-chain fatty acid uptake and metabolism [5]. Fatty acid biosynthesis is modulated by liver X receptor-α (LxRα) and its gene target, sterol regulatory element binding protein-1c (SREBP-1c) [6]. LxRα triggers the SREBP-1c expression, thus increasing the activity of genes involved in glycolysis and lipogenesis [7]. In addition, SREBP-1c is stimulated by the nuclear receptor heterodimer, which modulates genes such as acetyl-coenzyme (Co), A carboxylase (ACC), and fatty acid synthase (FAS) [8]. Moreover, the adipogenic transcription factor peroxisome proliferator-activated receptor gamma (PPARγ) is a direct target of SREBP-1c [9], and thus SREBP-1c has been revealed to have a vital role in adipocyte differentiation [10].

Impaired nutrient absorption leading to malabsorption and insufficient energy intake may result in dysfunction of liver lipogenesis [11]. Therefore, many dietary supplements can use in animals and humans to regulate nutrient absorption and improve energy metabolism. Boron (B), an essential element for humans and animals, has positive effects on reproduction, development, bone metabolism, hormonal activity, liver functions, brain activity, and different types of cancer [12]. Boron may promote the proliferation of intestinal epithelial cells and improve villi development, thus increasing nutrient absorption levels [13,14] and boosting nutrient digestibility in animals [12]. Besides its positive effects on intestinal health, B may be essential in regulating energy and lipid metabolism by controlling insulin release and contributing to energy substrate metabolism [15].

Boric acid [B(OH)_3_, BA] is widely used to investigate the mechanism of action of B in different biological systems [16,17]. However, the effect of sodium pentaborate pentahydrate (NaB_5_O_8_.5H_2_O, SPP) in biological systems is still unclear. SPP is generated from BA and sodium borate (borax), commonly used boron sources [18]. Previous studies have shown that SPP has osteogenic [19], wound healing [20], and antiobesity effects [21]. SPP administration may reduce white adipose tissue and liver weight and downregulate mouse adipogenic genes [21]. However, the role of boron, especially SPP form, in regulating intestinal nutrient transporters and hepatic lipid metabolism-related genes has not been studied. Based on the foregoing, the purpose of the present study was to clarify the effects of different B sources, especially SPP, on nutrient digestibility and expression of intestinal nutrient transporters, including GLUT1, GLUT2, GLUT5, SGLT1, FATP1, and FATP4 and liver lipid metabolism-related genes (LxRα, SREBP-1c, FAS, and PPARγ) in rats.

## 2. Materials and methods

### 2.1. Animals

In this study, twenty-one Wistar albino eight-week-old male rats (weighing 180 ± 20 g) were used. The total sample size was estimated by using G*power program (Version 3.1.9.3, Heinrich-Heine-Universität Düsseldorf, Germany) (N = 21, 1-β = 0.8, effect size = 0.75, α = 0.05). The rats were housed in plastic cages with free access to water and a rat chow diet (AIN-93M with minor modification, Table 1) under typical environmental conditions (12/12 h light/dark cycle, 22 °C, and 55% ± 3% humidity). The study was authorized by the Bingöl University Animal Experiments Local Ethics Committee (2019-06-03) according to EU Directive 2010/63/EU for animal research and performed in the Experimental Research Center of Bingöl University (BUDAM).

### 2.2. Experimental design

After one week of acclimatization, the rats were randomly divided into three groups (n = 7) as follows: (i) Control, which consisted of rats fed a chow diet and treated with 1 mL tap water as a vehicle via oral gavage (16-gauge needle, 76 mm); (ii) Sodium pentaborate pentahydrate (SPP), which consisted of rats fed a chow diet and supplemented with SPP; and (iii) Boric acid (BA), which consisted of rats fed a chow diet and supplemented with BA. Except for the amount of B received from the chow diet (AIN-93 diet contains 0.5 mg/kg B) [22], 8 mg elemental B/kg BW was administrated via oral gavage every other day for 12 weeks from SPP (45.2 mg/kg BW) and BA (43.7 mg/kg BW). SPP and BA contain 17.7% and 18.3% elemental boron (National Boron Research Institute-BOREN, Ankara, Turkey). The B dose was selected based on a previous study by Ergul et al. [23].

### 2.3. Sample collection

In the last five consecutive days of the experiment, fresh feces from individually caged rats were collected and pooled. The nutrient digestibility of rats in each group was measured using the indigestible indicator (chromium oxide, Cr_2_O_3_, 0.20%) [24]. At the end of the study, all rats were decapitated by cervical dislocation under xylazine (10 mg/kg) and ketamine hydrochloride (50 mg/kg) anesthesia. Serum (at least 2.5 mL), whole liver, and jejunum (5 cm) samples were removed immediately and kept at −80 °C for further analyses.

### 2.4. Nutrient digestibility

The collected fecal samples were dried in an oven at 60 °C for 72 h, while the feed samples were dried at 105 °C for 8 h. All fecal and feed samples were analyzed using AOAC standard techniques to evaluate the dry matter (DM), organic matter (OM), ash, crude protein (CP), and ether extract (EE) [25]. The samples were dried at 105 °C overnight in a forced air oven to determine DM (# 934.01). Ash was determined using a muffle furnace at 550 °C (# 942.05). The CP and EE were analyzed according to Kjeldahl (# 954.01) and Soxhlet methods (# 920.39), respectively. Chemical analyses were repeated at least three times. Nutrient digestibility was calculated using the following formula:


100-[100×(Cr in feed%/Cr in fecal samples%)×(nutrient in fecal samples%/nutrient in feed%)].

### 2.5. Mineral components analysis

The feed/fecal Cr levels were measured by atomic absorption spectrometry equipped with a graphite furnace (AAS, PerkinElmer, Analyst 800, Norwalk, USA). For this purpose, 0.3 g feed and feces samples were transferred into a Teflon digestion vessel and digested with 5 mL nitric acid (65%, Merck, Darmstadt, Germany) in the Berghoff microwave digestion system (Speedwave TM MWS-2, Eningen, Germany) with a three-step program. For calibration, six standard dilutions (0.5, 1.0, 2, 4, 8, and 10.0 μg/L) were prepared from Cr stock solution (Merck, Darmstadt, Germany). After digestion, the sample’s Cr levels were measured at a wavelength of 357.9 nm. The precision of quantitative measurements of Cr was confirmed by simultaneous analysis of certified reference material (bovine liver NIST® SRM® 1577c, New Jersey, USA), which was digested identically to the samples. For Cr, the mean sample recoveries were 98.7%.

The samples were digested using the Berghoff microwave digestion equipment for serum B analysis. For digestion, serum samples (0.5 mL) were heated to 250 °C in the microwave with 2 mL of concentrated HNO_3_ and 1 mL of 30% (v/v) H_2_O_2_. The samples were then transferred to 50 mL volumetric flasks and filled to the necessary volume with 18.2 MΩ/cm deionized water after cooling to room temperature. B levels were measured by flame atomic absorption spectrometry (FAAS, PerkinElmer, Analyst 800, Norwalk, USA) at a wavelength of 249.8 nm. Certified reference material (NIST® SRM® 3107, Merck, Darmstadt, Germany) was used for B analysis. The recovery was 89.9% [26].

### 2.6. Biochemical analyses

The BUN levels, ALT, and AST activities in serum were determined using biochemical kits by the LABGEO veterinary serum auto-analyzer (Samsung LABGEOPT10, Seoul, Korea).

### 2.7. Nutrient transporters and lipid-regulated transcription factors analyses

The real-time quantitative PCR method was used to determine mRNA nutrient transporters and lipid-regulated transcription factors expressions. The jejunum and liver samples were homogenized according to commercial RNeasy Mini kits (Qiagen, California, USA), following the manufacturer’s extraction guidelines. The amounts of total RNA in the homogenates were determined by a micro-volume spectrophotometer (MaestroNano, Maestrogen Inc., Hsinchu, Taiwan). cDNA was synthesized with TaqMan Reverse Transcription protocol using 2 μg of RNA. The synthesized cDNA was added to an SYBR Green PCR Master Mix (Catalog no. 330620, Qiagen) and used for the RT-qPCR. Expression of target genes (GLUT1, GLUT2, GLUT5, SGLT1, FATP1, PPARγ, SREBP-1c, LxR-α, and FAS) was assessed by Rotor-Gene Q (Qiagen, Maryland, USA). PCR reaction (denaturation: 95 °C, 15 s, 40 cycles; annealing: 60 °C, 15 s; extension: 70 °C, 30 s) was performed with 5 μL SYBR green master mix, 2 μL cDNA, 1 μL RNA-free water, and 2 μL primer pair (Table 2). Each PCR was performed at least three times. The mean Ct value of PCR reactions was considered for statistical analysis. GAPDH was used as the endogenous gene to standardize mRNA expressions. The expression levels of target genes were normalized according to the control group.

### 2.8. Statistical analysis

The data were analyzed using a statistical package tool (IBM Corp. 2012. Version 22.0. Armonk, NY, USA). The normality of the data was controlled with the Shapiro-Wilk test, and the homogeneity of variance was determined with the Levene test. For statistical comparisons, one-way analysis of variance (ANOVA) and Tukey’s post hoc test were performed. p < 0.05 is regarded as significant. Data demonstrated as mean ± standard deviation.

## 3. Results

### 3.1. Nutrient digestibility

**Figure 1:**
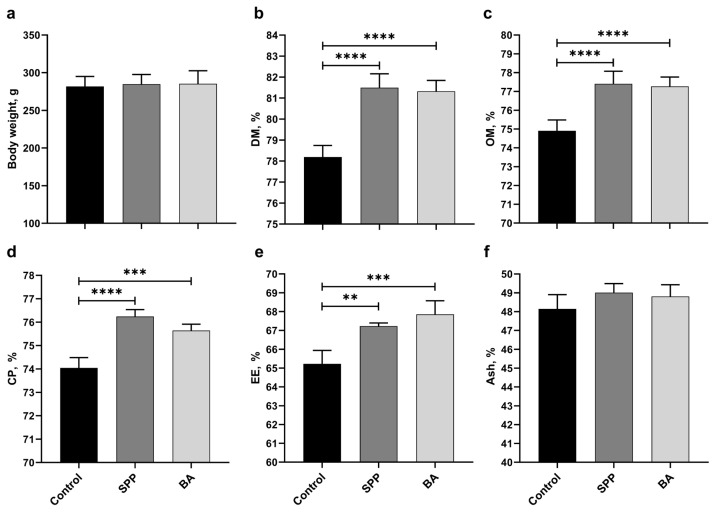
Effects of different B derivatives on body weight (a) and DM (b), OM (c), CP (d), EE (e), and ash (f) digestibility in rats. Data are shown as mean ± standard deviation. The difference between groups is indicated with asterisks (**p < 0.01, ***p < 0.001, and ****p < 0.0001). One-way ANOVA and Tukey’s post hoc tests were used for statistical comparisons.

B supplementation did not affect the final body weight of the rats (Figure 1a; p > 0.05). The SPP and BA groups had higher DM (p < 0.0001, for all), OM (p < 0.0001, for all), CP (p < 0.001 for the BA group and p < 0.0001 for the SPP group), and EE (p < 0.001 for the BA group and p < 0.01 for the SPP group) digestibility (Figures 1b–1e) than the control group. However, B supplementation did not affect ash digestibility (Figure 1f; p > 0.05).

### 3.2. Biochemical parameters

**Figure 2:**
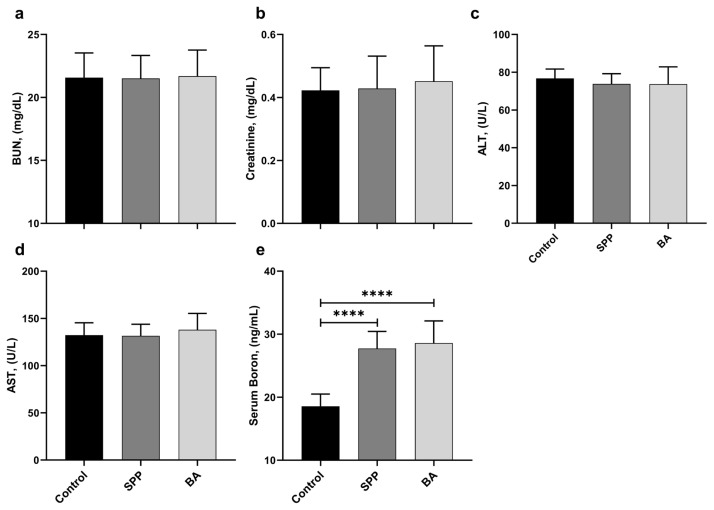
Effects of different B derivatives on serum BUN (a), creatinine (b), ALT (c), AST (d), and boron (e) levels in rats. Data are shown as mean ± standard deviation. The difference between groups is indicated with asterisks (*p < 0.05, **p < 0.01, and ****p < 0.0001). One-way ANOVA and Tukey’s post hoc tests were used for statistical comparisons.

There was no statistical difference in serum BUN, creatinine, ALT, and AST parameters between the control and other groups (Figures 2a–2d, p > 0.05). The SPP (27.73 ng/mL) and BA (28.59 ng/mL) groups had higher serum B levels than the control group (18.56 ng/mL) (Figure 2b; p < 0.0001, for all). However, the serum B level was increased in the SPP and BA groups compared to the control group (p < 0.0001), and there was no statistical difference between the SPP and BA groups (p > 0.05).

### 3.3. Intestinal nutrient transporters

**Figure 3:**
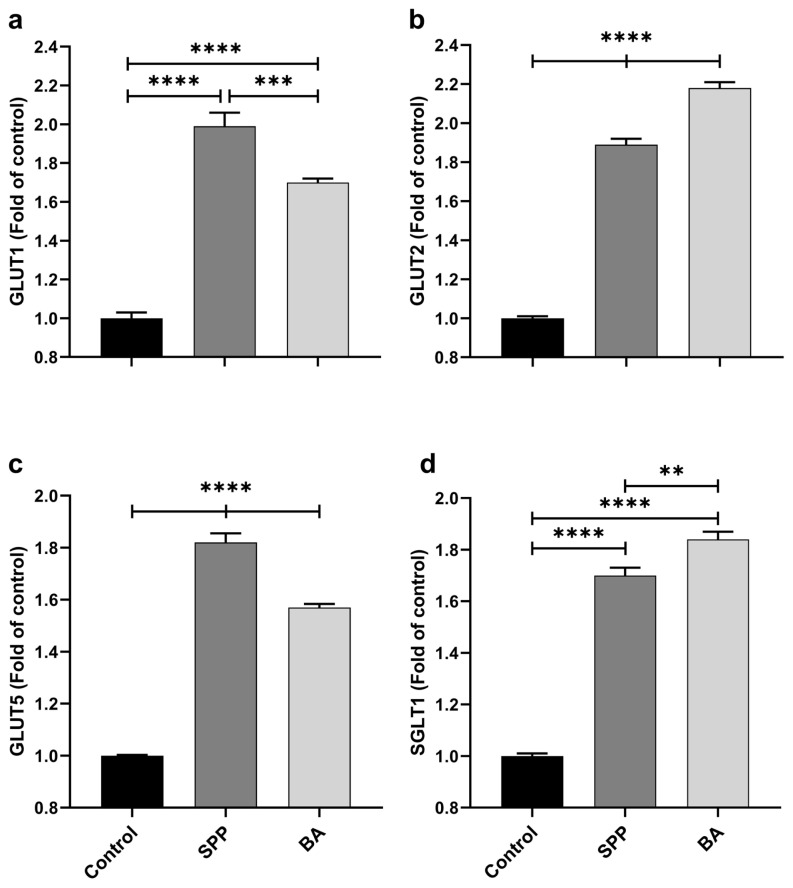
Effects of different B derivatives on jejunal GLUT1 (a), GLUT2 (b), GLUT5 (c), and SGLT1 (d) expression levels in rats. Each PCR was performed at least three times. GAPDH was used as the endogenous control gene. The expression of target genes was normalized to the control group. Data are shown as mean ± standard deviation. The difference between groups is indicated with asterisks (*p < 0.05, **p < 0.01, ***p < 0.001, and ****p < 0.0001). One-way ANOVA and Tukey’s post hoc tests were used for statistical comparisons.

**Figure 4:**
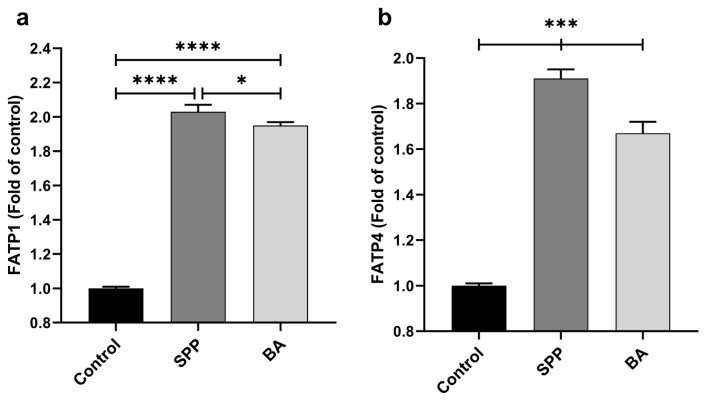
Effects of different B derivatives on jejunal FATP1 (a) and FATP4 (b) expression levels in rats. Each PCR was performed at least three times. GAPDH was used as the endogenous control gene. The expression of target genes was normalized to the control group. Data are shown as mean ± standard deviation. The difference between groups is indicated with asterisks (*p < 0.05 and ****p < 0.0001). One-way ANOVA and Tukey’s post hoc tests were used for statistical comparisons.

Rats administrated SPP and BA had higher jejunal GLUT1, GLUT2, GLUT5, SGLT1, FATP1, and FATP4 mRNA expression levels than nonsupplemented rats (Figure 3 and 4; p < 0.0001, for all). GLUT1 and GLUT5 expressions were significantly higher in the SPP group than in the BA group (p < 0.0001). However, GLUT2 and SGLT1 expressions were increased in the BA group compared to the SPP group (p < 0.0001 for GLUT2; p < 0.01 for SGLT1). Additionally, SPP administration effectively stimulated the jejunal FATP1 (Figure 4a, p < 0.05) and FATP4 (Figure 4b, p < 0.0001) expression compared to BA administration.

### 3.4. Liver lipid-regulated transcription factors

**Figure 5:**
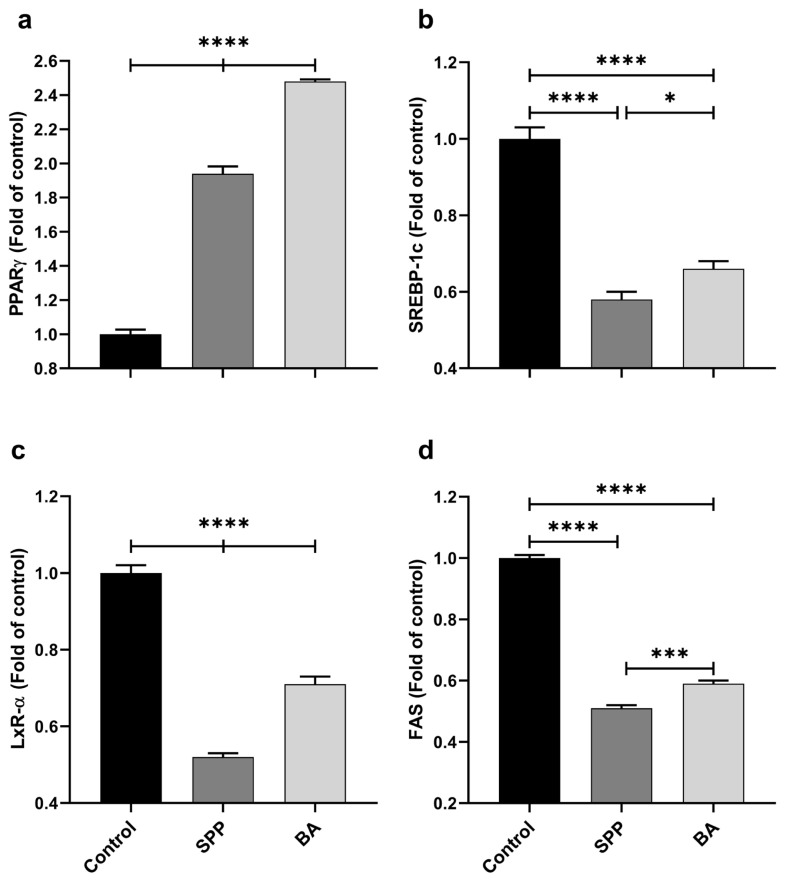
Effects of different B derivatives on liver PPARγ (a), SREBP-1c (b), LxR-α (c), and FAS (d) expression levels in rats. Each PCR was performed at least three times. GAPDH was used as the endogenous control gene. The expression levels of target genes were normalized to the control group. Data are shown as mean ± standard deviation. The difference between groups is indicated with asterisks (*p < 0.05, **p < 0.01, ***p < 0.001, and ****p < 0.0001). One-way ANOVA and Tukey’s post hoc tests were used for statistical comparisons.

BA-supplemented rats had significantly higher liver PPARγ expression levels than other groups (Figure 5a, p < 0.0001). Liver SREBP-1c (Figure 5b) and FAS (Figure 5d) expression levels were markedly decreased by SPP supplementation compared to nonsupplemented (p < 0.0001, for all) and BA-supplemented rats (p < 0.05 for SREBP-1c and p < 0.001 for FAS). Similarly, LxR-α (Figure 5c) expression was significantly inhibited in the SPP group compared to the other groups (p < 0.0001 for all).

## 4. Discussion

The present study showed that SPP and BA could enhance nutrient digestibility and intestinal glucose and fatty acid transporters, as well as regulate hepatic lipogenesis. B is absorbed from the gastrointestinal tract and is rapidly distributed into tissues [27,28]. This rapid action of B probably increased serum B levels with SPP and BA supplementation in this study. Because 90% of the absorbed B is excreted in the urine within a few days, it may not cause toxicity and does not accumulate in soft tissues when consumed in low doses [12]. In the current study, we observed that a dose of 8 mg/kg elemental B administration for 12 weeks did not lead to toxicity in rats and did not negatively alter the serum BUN, ALT, AST, and creatinine levels.

Previous studies have shown the positive effects of BA on DM, OM, CP, EE, and ash digestibility [13,14]. Cho et al. showed that BA added to the diet (equal to 5 mg/kg dietary elemental B) reduced the incidence of diarrhea while increasing nutrient and energy digestibility in pigs [13]. Vijay Bhasker et al. reported that 352 mg/kg dietary sodium borate supplementation (equal to 40 mg/kg dietary elemental B) positively influenced nutrient digestibility in rats [29]. Our results demonstrated for the first time that SPP and BA supplementation have similar effects on nutrient digestibility in rats. Likely, the positive impact of SPP on nutrient digestibility seems to be related to cell proliferative activity [30], as does BA [13,14]. Also, the water solution of BA [31] and SPP [32] has a low buffering capacity and almost the same pH values as the intestinal tract; thus, due to these boron compounds did not decompose to borate anions at physiological pH [33], they may have exerted the same effect on nutrient digestibility. However, the lack of assessment of the histopathological alterations and luminal pH measurements is the limitation of our study.

Previous studies reported that BA might promote cell proliferation in intestinal epithelia, increase villus height and crypt depth [14,34], and improve intestinal health by modulating intestinal permeability [35]. Thus, in the current study, the increased expression levels of glucose and fatty acid transporters may have stemmed from boosted nutrient digestibility [36] and improved intestinal integrity, as reported by previous studies, after B supplementation [14,34,35].

To the best of our knowledge, this is the first study to evaluate the effect of the SPP and BA treatment on GLUT1, GLUT5, FATP1, and FATP4 expressions in the jejunum of rats. Parallel to the nutrient digestibility results, in the present study, rats treated with SPP had higher expression of GLUT1, GLUT5, FATP1, and FATP4 in the jejunum of rats treated with BA. Additionally, the GLUT2 and SGLT1 expression in the jejunum of rats was much higher in groups supplemented with BA compared with the SPP. No previous studies in jejeunum investigate the effects of SPP and BA supplementation on the nutrient transport response with which to compare this study. However, it has been reported that GLUT1 is the primary mediator of cellular glucose influx and cell proliferation, among other GLUTs [37]. Thus, intestinal GLUT1 expression might have been elevated by SPP owing to its higher cell proliferating ability, as reported before [20,30]. Also, an in vitro study by Aydın et al. declared that 19.5 μg/mL of BA treatment could relatively increase the GLUT2 expression than SPP in pancreatic β-cells [38].

In the present study, SPP and BA supplementation increased PPARγ and decreased SREBP-1c, LxR-α, and FAS expression in the liver of rats. These data are consistent with earlier reports showing the effects of boron on lipid metabolism-related genes [21,39]. For example, Kucukkurt et al. showed that 10 mg/kg B (as BA) increased PPARγ expression in the liver in HFD-fed rats [39]. On the other hand, supplemental BA (30 mg/L B) in drinking water decreased the PPARγ in the liver [40]. More recently, Abdik et al. found that oral 500–1500 mg/kg SPP administration reduced the PPARγ, SREBP1, and FAS mRNA expression and adipogenesis-related genes in the white adipose tissue in HFD-fed diabetic mice [21]. Although there are no data regarding the effect of B compounds on LxR-α, it is well known that LxR-α can stimulate the SREBP-1c and FAS activity in the liver and increase lipid accumulation [41]. Additionally, while previous studies have shown that B may be effective in suppressing liver adipogenesis [21,39,40], these studies did not demonstrate the molecular mechanism of action of B on the LXR-α/SREBP-1c/FAS cascade in rat liver. Therefore, we show here for the first time that B can regulate lipogenesis in rat liver by inhibiting the LxR-α and possibly the LXR-α/SREBP-1c/FAS cascade. In addition, SPP form inhibited the expression of SREBP-1c, LxR-α, and FAS in the liver of rats more potently than BA. Similarly, Yuksel et al. demonstrated that SPP promotes hair growth in rats by stimulating the Wnt/β-catenin pathway [42] that inhibits SREBP-1c [43], LxR-α [44], and FAS [45].

## 5. Conclusions

The present data showed that SPP and BA improved lipid metabolism and intestinal health by modulation of liver lipid-related genes and intestinal nutrient transporters, including glucose and fatty acid transporters. The efficacy of boron as SPP was more notable than boron as BA, which could be attributed to higher bioavailability. However, clinical studies in humans and animals are needed to support current findings. Therefore, these data may shed light on future studies using boron (especially SPP) to prevent gastrointestinal and lipid metabolism disorders.

## Figures and Tables

**Table 1:** Composition of rat chow diet (AIN-93M).*

Ingredients	%
Casein	20.00
Starch	57.95
Sucrose	5.00
Soybean oil	7.00
Cellulose	5.00
Mineral premix**	3.50
Vitamin premix***	1.00
l-cysteine	0.30
Choline bitartrate	0.25
**Chemical analysis**	
Metabolic energy, MJ/kg	15.93
Crude protein, %	17.90
Ether extract, %	7.00
Ash, %	4.20
Calcium, g/kg	5.00
Phosphorus, g/kg	3.00

* AIN-93M diet contains 0.5 mg/kg elemental boron. Each rat intakes 0.001 mg (0.5 mg/1000 g diet) of elemental boron from the chow diet.

** AIN-93G-MX

*** AIN-93G-VX

**Table 2:** RT-qPCR primers.

Gene Name	Accession Number	Forward	Reverse
GLUT1 (SLC2A1)	NM_138827.2	TCTCTGTCGGGGGCATGATT	AACCCATAAGCACGGCAGAC
GLUT2 (SLC2A2)	NM_012879.2	AGTCACACCAGCACATACGA	TGGCTTTGATCCTTCCGAGT
GLUT5 (SLC2A5)	NM_019741	GAAGACACACTGAGCCGTGGA	CCTTTCTTCAGCAGGGAAGTGTC
SGLT1 (SLC5A1)	NM_013033.2	AAGCGATTTGGAGGCAAGCG	CCAGTCCCCCTGTGATGGTG
FATP1 (SLC27A1)	NM_053580.2	TGCGAGAACCCGTGAGGAA	CGATACGCAGAAAGCGCCAG
FATP4 (SLC27A4)	NM_001100706.1	GGGTGCCAACAACAAGAAGATTGC	TGCGGTCTCGGAAGTACAGGTAG
PPARγ	NM_013124.3	GACCTGAAGCTCCAAGAATACCA	CCCACAGACTCGGCACTCA
SREBP-1c	NM_001276708.1	GACGACGGAGCCATGGATT	GGGAAGTCACTGTCTTGGTTGTT
LxR-α	NM_031627.2	CCTGATGTTTCTCCTGACTC	TGACTCCAACCCTATCCTTA
FAS	NM_017332.2	CCACCCTGTAGGTCACCGTTT	GTGGGTATAAGCGTTCAGCTGC
GAPDH	NM_017008.4	GGTTACCAGGGCTGCCTTCT	CTTCCCATTCTCAGCCTTGACT
